# Evaluation of a wetland classification system devised for management in a region with a high cover of peatlands: an example from the Cook Inlet Basin, Alaska

**DOI:** 10.1007/s11273-016-9504-0

**Published:** 2016-10-19

**Authors:** Michael Gracz, Paul H. Glaser

**Affiliations:** 1 Conservation Sciences Program, University of Minnesota, Saint Paul, MN USA; 2 Department of Earth Sciences, University of Minnesota, Minneapolis, MN USA

**Keywords:** Wetland classification, Boreal peatlands, Hydrogeomorphic, Southcentral Alaska, Cook Inlet, Wetland functions, Multi-response permutation procedure

## Abstract

Several wetland classification schemes are now commonly used to describe wetlands in the contiguous United States to meet local, regional, and national regulatory requirements. However, these established systems have proven to be insufficient to meet the needs of land managers in Alaska. The wetlands of this northern region are predominantly peatlands, which are not adequately treated by the nationally-used systems, which have few, if any, peatland classes. A new system was therefore devised to classify wetlands in the rapidly urbanizing Cook Inlet Basin of southcentral Alaska, USA. The Cook Inlet Classification (CIC) is based on seven geomorphic and six hydrologic components that incorporate the environmental gradients responsible for the primary sources of variation in peatland ecosystems. The geomorphic and hydrologic components have the added advantage of being detectable on remote sensing imagery, which facilitates regional mapping across large tracts of inaccessible terrain. Three different quantitative measures were used to evaluate the robustness and performance of the CIC classes relative to that of other commonly used systems in the contiguous United States. The high within-group similarity of the classes identified by the CIC was clearly superior to that of the other systems, demonstrating the need for improved wetland classification systems specifically devised for regions with a high cover of peatlands.

**Electronic supplementary material:**

The online version of this article (doi:10.1007/s11273-016-9504-0) contains supplementary material, which is available to authorized users.

## Introduction

Peatlands cover approximately 20 % of all boreal landscapes (Vitt 2006) including the lowlands of the Cook Inlet Basin (CIB) in southcentral Alaska. This 102,000 km^2^ basin, at the northern reaches of Pacific Ocean, contains a mosaic of relatively pristine uplands and wetlands including spawning habitat for healthy populations of all five species of Pacific salmon. Peatlands within the CIB lowlands contribute to stream flows and thereby help to maintain fluvial habitats required for the survival of healthy salmon stocks (Gracz et al. 2015). These peatlands also provide a variety of other ecosystem services that affect human population centers such as flood regulation, recreational opportunities, and water purification (Millennium Ecosystem Assessment 2005). As the population of CIB and Alaska continues to grow so has the need to implement a classification system for use in wetland assessment that emphasizes the linkages between the hydrogeologic settings of oligotrophic peatlands and their functions and services.

Land managers need a classification system that can be used to classify oligotrophic peatlands, which have been generally differentiated into discrete types based on landform patterns or hydrochemical properties (Gore 1983; Zoltai et al. 1988; Charman 2002; Weider et al. 2006; Rydin and Jeglum 2006). In practice however, the classification and mapping of peatlands is constrained by site access limitations. In regions where wetlands cover large areas and the road network is sparse, remote sensing provides the best means to scale up local field measurements to the level of the regional landscape. Although a classification system could be devised based on the hydrochemical factors that determine the potential directions of peatland development in such regions, this type of classification system would require an impractical degree of field sampling to implement. For example, the calcium concentration and pH of surface waters have been linked to the different types of peatlands in boreal regions (e.g. Weber 1902; Sjörs 1950; Glaser et al. 1981; Rydin and Jeglum 2006; Wieder et al. 2006; Ye et al. 2012). However, unless these hydrochemical indicators can be inferred from proxy evidence that is visible on remote sensing imagery they will have limited value for mapping peatland types across any broad region.

Alternatively, the geomorphic setting and hydrology of a wetland can be used in conjunction with remote sensing imagery to establish an effective wetland classification scheme for remote regions with limited road access. Geomorphic and hydrologic factors have been recognized as fundamental variables that are intrinsically related to wetland functions (Brinson 1993). The geomorphic setting of a peatland imposes physical constraints on hydrologic flow systems and also provides sources for the dissolved mineral solutes that have long been recognized as the fundamental factor responsible for the different types of peatlands (Weber 1902; Du Rietz 1949; Kulczyński 1949; Sjörs 1950; Glaser et al. 1990). Striking landform patterns on peatlands have been successfully used to define the most important types of bog and fen on remote-sensing imagery (e.g. Sjörs 1963; Glaser et al. 1981; Glaser et al. 2004; Siegel and Glaser 1987).

A fundamental hydrologic factor controlling the patterning of peatland vegetation is the average elevation of the water table (Sjörs 1950b; Malmer 1986; Foster et al. 1988; Waddington and Roulet 2000). For example, in northern Minnesota and in the Hudson Bay Lowlands, indirect gradient analyses first aligned vegetation samples according to the elevation of the water table, with the inundated flark plots, at one end of the ordination and the better-drained forested plots at the other (Glaser 1992; Glaser et al. 2004). Even though the importance of these two fundamental factors of hydrology and water chemistry are widely recognized in the peatland literature, they have not been explicitly incorporated into wetland classification systems currently in use in the U.S.A.

Two classification systems are widely used across the U.S.A.: the National Wetlands Inventory (NWI) and the HGM classification system (Brinson 1993; Smith et al. 1995). NWI (Cowardin et al. 1979) does not differentiate among peatland classes because peatlands are uncommon except in a few northern states such as Alaska (Kivinen and Pakarinen 1981). The classification system of the hydrogeomorphic model (HGM) of wetland functional assessment uses hydrologic and geomorphologic factors to distinguish among wetlands, but this system only has a single class exclusively for peatlands (Smith et al. 1995). Further, when local managers attempted to classify peatlands in the CIB to develop an HGM guidebook for a representative class, they recognized that the seven national-level classes of the HGM system did not adequately represent the common peatlands in this region. The HGM class *Organic Soil Flat,* for example, is described as bogs with predominantly vertical hydrodynamics, whereas the broad class *Slope wetlands* includes fens defined as having horizontal hydrodynamics (Smith et al. 1995). However, peatlands frequently comprise a mosaic of bogs and fens exhibiting complex interacting hydrodynamics along both horizontal and vertical flow vectors (Ingram 1983; Siegel and Glaser 1987; Siegel et al. 1995; Reeve et al. 2001; Glaser et al. 2004; Spence et al. 2011). As a compromise, (Hall et al. 2002) hybridized the two classes into a Slope/Flat type while developing the guidebook. However, a single class is insufficient to distinguish important functional differences among the diverse peatland complexes in CIB that have important implications for wetland assessment and management.

### Objectives

Here we describe the development of the Cook Inlet Classification (CIC) system, which was specifically designed to distinguish the principal types of oligotrophic peatlands in the CIB, and then use a multivariate analysis to evaluate the within-group similarity of the classes of the CIC. Because multivariate analysis has a limited capacity for statistical inference, standards of comparison are needed to compare the relative robustness of the classes. For this comparison, the within-group similarity of the classes distinguished by the CIC system is compared to the within-group similarity produced by three other wetland classification systems: (1) NWI (Cowardin et al. 1979), (2) the Landscape position, Landform, Water flow path, and Waterbody system (LLWW), which was developed in the glaciated northeastern region of the USA for use with NWI (Tiner 2011), and (3) NWI + LLWW, a combination of 1 and 2 above (Brooks et al. 2011). If the CIC system produces a within-group similarity surpassing that of the other systems, then it should provide a useful foundation for managers in the region while also providing insights on the relationship between the fundamental factors controlling ecosystem function and the hydrogeologic setting of oligotrophic peatlands.

### Study area

**Fig. 1:**
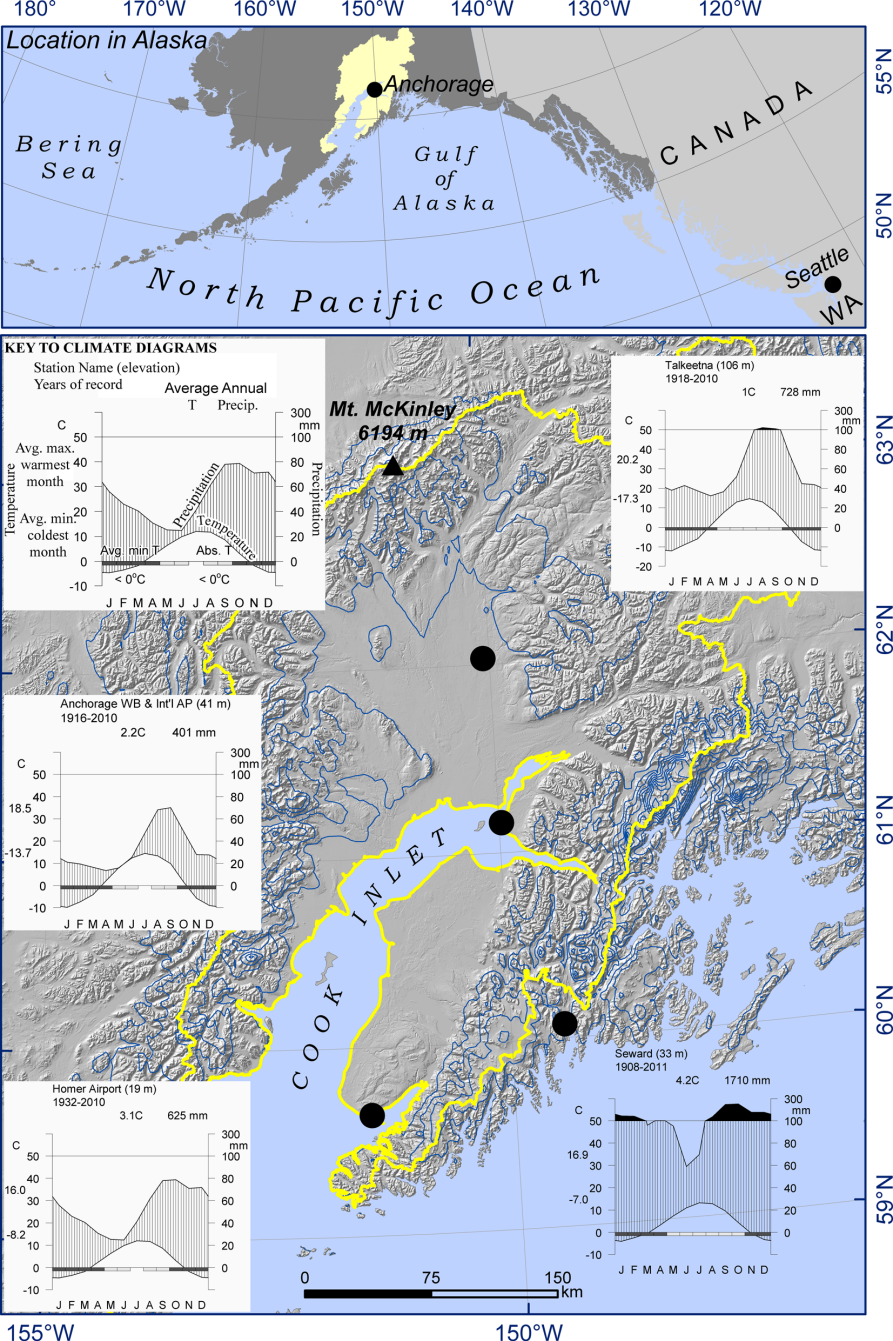
Location, physiography, and climate of Cook Inlet Basin, Alaska, shown by *yellow polygon* (*top*) and outlined in *yellow* (*bottom*). *Blue lines* show 1000 mm isohyets (*bottom*). *Black circles* on *bottom* map show locations of climate stations. Climate diagrams follow Walter and Leith (1960)

Cook Inlet Basin (CIB), Alaska is centered at 151° W longitude between 59°N and 63°N latitude and drains to Cook Inlet, a large marine embayment formed in a rapidly subsiding fore-arc basin (Hartman et al. 1971). The 101,635 km^2^ basin is surrounded by numerous glaciated mountainous terranes of diverse lithology (Silberling et al. 1994), including the highest point in North America (Fig. 1). The lowland portion of the Basin is composed of sediments of Paleogene to Neogene age (65.5–2.6 mya) up to 8700 m thick (Hartman et al. 1971) derived from the surrounding diverse lithologies of the mountainous terranes including: sandstone, arkose, argillite, greywacke, slate, granodiorite, breccia, and intermediate-to-felsic volcanic rocks (Beikman 1994). Pleistocene epoch (11.7 ka–2.65 ma) alpine glaciations originating in the diverse mountain lithologies mantled the sedimentary rocks with deposits up to 2800 m thick (Freethey and Scully 1980), producing a geomorphologically complex landscape. The complexity of this landscape was further increased for at least the past 10.5 ma by active volcanos along the western margin of CIB that have blanketed the entire basin with volcanic ash of diverse composition, including calc-alkaline dacite and basaltic andesite (Fournelle et al. 1994; Riehle 1985).

The physiography of the CIB basin supports a strong but complex maritime-to-continental climatic gradient. Winter temperatures always fall below −40 °C in the interior, while at coastal stations they rarely reach −20 °C (Fig. 1). Annual precipitation ranges from 300 to 1000 mm in the lowlands, and can be as high as 9000 mm at glaciated mountain passes (PRISM Climate Group 2011; Online Resource). Nearly half of the annual precipitation falls from September–December, whereas less than 20 % falls from April through July (Utah Climate Center 2013). Evapotranspiration can exceed precipitation in a small area of rain shadow formed by the surrounding mountains. Wetlands still occur in this pocket of moisture deficit because recharge in the surrounding mountains, where precipitation far exceeds evapotranspiration, is rapidly transmitted to the lowlands through permeable bedrock and glaciofluvial deposits (Jokela et al. 1991; Kikuchi 2013). Over most of the lowlands, however, precipitation is sufficient to maintain waterlogged soils in a range of physiographic settings (e.g. depressions, watershed divides, and seepages at the slope breaks) and approximately 20 % of the lowland surface is covered by peatlands.

## Methods

### Cook Inlet Classification system

The Cook Inlet Classification (CIC) system has been used to map 1508 km^2^ of peatlands over an area of 7589 km^2^ (Online Resource). The system is comprised of geomorphic and hydrologic classes that are readily detectable on stereo-paired aerial photographs or in combination with shaded-relief images of digital elevation models derived from high-resolution light detection and ranging (LiDAR) data. Wetlands underlain by mineral substrates are also described in the CIC, such as tidal and floodplain wetlands. A complete guide to all the wetland types mapped in the CIB is presented in a digital file, which contains a detailed map, legend, idealized cross-sectional diagrams of plant associations, and graphical summaries of climatic, water chemistry, and plant prevalence index data (Online Resource).

The CIC uses seven Geomorphic Components and six Hydrologic Components to distinguish the different classes describing oligotrophic peatlands. The Geomorphologic Components of the CIC system were developed using an iterative approach guided by the regional geologic literature, soil maps, field observations, and discussions with regional experts. These components were designed to capture the principal hydrogeologic settings of wetlands within the Cook Inlet Basin that impose geomorphic constraints on hydrology and water chemistry. To identify which geomorphic features best defined distinct peatland classes, the peatlands in two small pilot areas were mapped and then sampled in the field to determine their hydrology (i.e. water table elevations), water chemistry (e.g. Ca concentrations, pH etc.), and vegetation assemblages. Different names for the Geomorphic Components were applied and adjusted until an adequate set of names were identified that separated peatland types with discrete hydrological, chemical, and botanical characteristics. After the pilot areas had been satisfactorily classified, new Geomorphic Components were added to the classification system as the mapping area expanded during subsequent years. Plant cover and water chemistry data were evaluated along with data from instrumented wells and piezometers to refine the names or support new designations.

This iterative approach was modified to account for the practical needs of regulatory jurisdiction and management. For example, kettle depressions are common features in many areas of the CIB. However, these kettles can be further characterized as: (1) closed basin, which is an important jurisdictional criterion for wetland permitting, (2) closed basin, but strongly connected to groundwater flow within a zone of moisture deficit, and (3) open basin, i.e. connected by surface water to a navigable water body. Although these three types of landforms could all be classified as kettles, they clearly lie in different hydrogeologic settings, some of which may not be subject to regulatory authority. Therefore, different Geomorphic Component names were assigned to each of them: (1) *Depressions* are kettles in closed basins (Neuendorf et al. 2005) lacking a strong hydrologic connection to groundwater discharge, (2) *Spring fens* are closed basins strongly connected to groundwater flow (Zoltai et al. 1988) in a zone of moisture deficit, or (3) *Kettles* are open basins (Table 1).

**Table 1:** The geomorphic components of freshwater peatlands in the Cook Inlet Classification

CIC geomorphic component	LLWW synonyms	Landform	Diagnostic characteristics in CIC
Depression	Terrene basin inflow Terrene basin inflow lotic fringe	Closed basin ice-block depression	Surrounded by upland, Precipitation > Evapotranspiration
Kettle	Terrene basin throughflow Terrene basin headwaters Lotic fringe throughflow	Open basin ice-block depression	Connected to navigable water by surface water or wetland
Spring fen	Terrene basin throughflow groundwater Lotic fringe	Closed basin ice-block depression	Surrounded by upland, P ≤ ET, in depressions fed by strong groundwater discharge
Headwater fen	Terrene basin outflow headwaters Terrene basin throughflow headwaters	Cirque	Headwater peatland of a first-order stream near or above treeline
Relict glacial drainageway	Terrene slope headwaters Terrene slope throughflow Lentic slope fringe	Abandoned or underfit stream valleys	Broadly linear features filled with peat, with or without modern stream channels
Relict glacial lakebed	Terrene slope throughflow	Extensive peatlands over proglacial lake deposits	Large, low-gradient peatlands
VLD trough	Lotic fringe throughflow Terrene slope throughflow	Valleys between “Very Large Dunes”^a^	Poorly understood ripple-like features in the Meadow Lakes area of the Matanuska Valley

All three of the NWI classes of PEM, PSS and PFO occur in all of the CIC classes

^a^As described by Wiedmer et al. (2010) in a paper proposing the genesis of the dunes by a late-Pleistocene megaflood

In the CIC, the Hydrologic Components are represented as numbered values describing the seasonal variability of water levels. Lower values represent wetlands with water levels at or above the land surface throughout the growing season, whereas higher values represent wetlands with more variable water levels that on average are deeper below the surface. Seasonal water-level variation was used to define Hydrologic Components because a large number of vegetation studies in a variety of settings, including peatlands, relate the first ordination axis of plant presence and abundance data to a moisture gradient (Bray and Curtis 1957; Curtis 1959; Whittaker 1970; Peet 1980; Kormárková 1980; Foster et al. 1988; Dunham 1989; Glaser 1992; Pinder and Rosso 1998; Glaser et al. 2004; Zelnik and Čarni 2008).

This study distinguished wetlands from uplands based on the criteria adopted by the Alaska Regional Supplement (USACE 2007) to the United States Army Core of Engineers delineation manual (Environmental Laboratory 1987). In general, these manuals define wetlands according to the relative persistence of the water table near the ground surface as a proportion of the growing season length. Because of the short growing season, CIB sites generally satisfy the wetland criteria in these manuals if they maintain water levels within 30 cm of the ground surface for approximately 2 weeks during the growing season (US Department of Agriculture 2011). However, the peatlands described here often support such elevated water levels throughout the growing season.

### CIC: class assignment

Wetland class assignments in the Cook Inlet Classification were initially made in the lab, guided by a variety of resources including geologic maps, soil maps, NWI mapping, and stereo-paired aerial photographs. Once made, a representative sample of the class assignments was subsequently corrected during site visits. Corrected CIC class names were matched (cross-walked) to LLWW class names in 2005 by R. Tiner, the developer of the LLWW, using descriptions written for the CIC (Gracz 2015). The five Landform classes of LLWW that matched CIC classes were: Terrane Slope, Basin, and Headwaters; and Lentic and Lotic Fringe. Landform classes were combined with three water flow-path classes: Throughflow, Inflow, and Outflow and were further refined by the modifiers Groundwater, Headwaters, and Lotic Fringe. Although much more complex names are possible in the LLWW system, we limited the names so that similar levels of classification could be compared across the systems. The limit further allowed us to evaluate the ability of a simple dichotomous naming system to create high within-group similarity based on relevant ecological measures.

The Palustrine System of NWI was assigned to each plot along with one of the three NWI plant physiognomic classes: emergent (PEM), shrub-scrub (PSS), and forested (PFO), using NWI maps (USFWS 2010). Brooks et al. (2011) suggest that combining a hydrogeomorphic model (HGM) classification system with the NWI system would produce a system emphasizing fundamental hydrogeomorphic characteristics built on existing NWI terminology. To compare CIC to such a classification scheme, we combined the NWI classes with LLWW names for each plot. Because the developers of each classification essentially made the class assignments, errors of misclassification are negligible. Although mapping errors are possible in NWI (Dvorett et al. 2012), we assigned classes based on the conditions found on the field visit.

### Field measurements

In the field we measured: plant cover by species, water level, specific conductance (SC), and pH in 222 plots within representative stands of vegetation that were stratified across the CIC peatland classes in proportion to their occurrence. Percent cover class was estimated for each plant taxon. All vascular taxa covering 1 % or more were identified at least to the species level. Cover classes were in 10 % categories, except between 1 and 7 % where one percent classes were used. Cover less than 0.5 % was tabulated as 0.1 %. Measurements of SC and pH were taken in surface water where available or in a shallow pit excavated no more than 30 cm deep. Measurements were made using a YSI 63 m, which was two-point calibrated for pH between each measurement and cleaned daily. Estimates were made of the depth of the water table below the surface at 957 plots sampled as part of this study and during the Western Kenai Soil Survey (VanPatten 2005) to calibrate a proxy for water-level variation.

### Plant prevalence index and detrended correspondence analysis

Two separate procedures were used to evaluate the within-group similarity produced by the CIC and the other common classification schemes. The first procedure used SC, pH, and plant prevalence index. Specific conductance should be strongly correlated with calcium concentration because calcium is typically the most abundant cation in natural surface waters. The calcium concentration and pH of peatland porewaters are the two chemical factors most closely related to plant distribution and other processes in oligotrophic peatlands (Weber 1902; Kuczyński 1949; Sjörs 1950; Glaser et al. 1981, 1990; Foster et al. 1988; Ye et al. 2012). Porewater chemistry reflects the relative influence of groundwater versus precipitation on peatland water supply in areas with similar underlying geology (Siegel and Glaser 1987; Hill and Siegel 1991; Siegel et al. 1995; Glaser et al. 2004). Moreover, SC and pH measurements can be collected efficiently over a broad area.

Plant prevalence index (PI) was used as a proxy for the seasonal variability of water levels in place of the single water depth measurement made at each site. A single measurement may not be a reliable indicator of water levels because such measurements may be biased by antecedent conditions without equilibrating to the seasonal average value. Further, reliable measurements of water levels throughout the season and over multiple years requires an impractical intensity of data collection in large regions with limited site access. The PI calculation uses the wetland indicator status of each plant in a plot as a criterion for the wetland definition in the Alaska Regional Supplement to the Delineation manual (USACE 2007) and in other regions (De Steven 2015). Wetland indicator status was assigned using the values in the PLANTS database (USDA 2010). The suitability of PI as a proxy for water level variability was examined by comparing PI to measurements of water table depth at 957 plots.

The second procedure evaluated the within-group similarity produced by each classification system using the axis scores from a detrended correspondence analysis (DCA) of plant cover data (Hill 1979; McCune and Mefford 1999). DCA is a modified reciprocal averaging technique that produces multiple axis scores for samples based on the presence and abundance of entities. It is appropriate for matrices with many zeros, such as found with plant cover data. The modification is the forced removal of the arch and higher-order polynomial relationships produced in the second and subsequent axes by reciprocal averaging (McCune and Mefford 1999). The scores for the first three axes of the DCA were used as explanatory variables.

### Multi-response permutation procedure

Multi-response permutation procedure (MRPP) was used in PC-ORD to assess within-group similarity (McCune and Mefford 1999). MRPP is a non-parametric procedure that produces a *P* value describing whether or not class assignments differ from random. With a large sample size, such as the CIB samples, *P* values are often statistically significant, and the challenge becomes the interpretation of the ecological significance of the non-random groupings. To assist with this ecological interpretation, MRPP produces an *A* value, the chance-corrected within-group agreement, which ranges from zero to one. When *A* = 1 all plots in each group are identical to each other. Values for *A* ≥ 0.1 can be ecologically meaningful, and *A* = 0.3 is “fairly high” according to McCune and Mefford (1999). In the procedure using PI, pH, and SC, the measurements were made commensurate by normalization (Mielke et al. 1981). This procedure was run with the CIC first using only its Hydrologic Components; second, using only its Geomorphic Components; and finally, with the complete classes, so that the relative contribution of each component could be evaluated separately.

## Results

### CIC classes

**Fig. 2:**
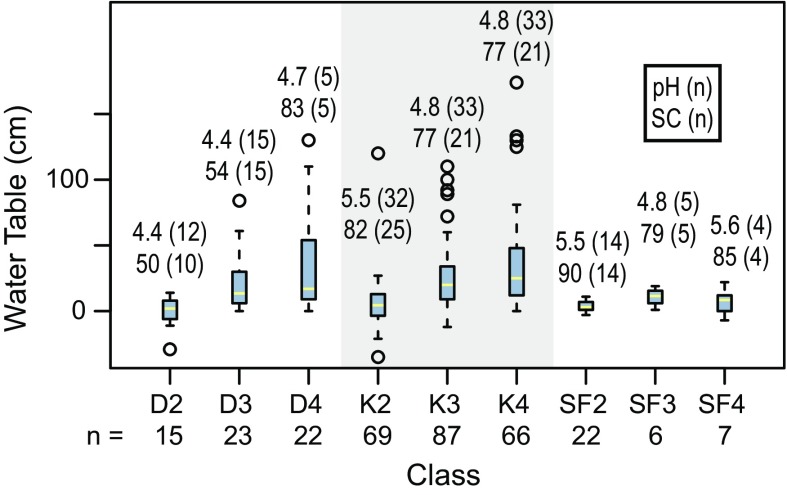
Water levels and chemistry of peatlands in kettle landforms. *D2*-*4* are *Depressions*, *K2*-*4* are *Kettles*, and *SF2*-*4* are *Spring Fens* in the Cook Inlet Classification system. *Blue boxes* enclose the inner two quartiles, the *yellow horizontal lines* inside the *boxes* are median values, and the *whiskers* extend to the last value within 1.5 times the inner quartile range. Values outside of the inner quartile are plotted as *circles*. Numbers are pH, specific conductance, and sample n according to the key in the *box*

**Fig. 3:**
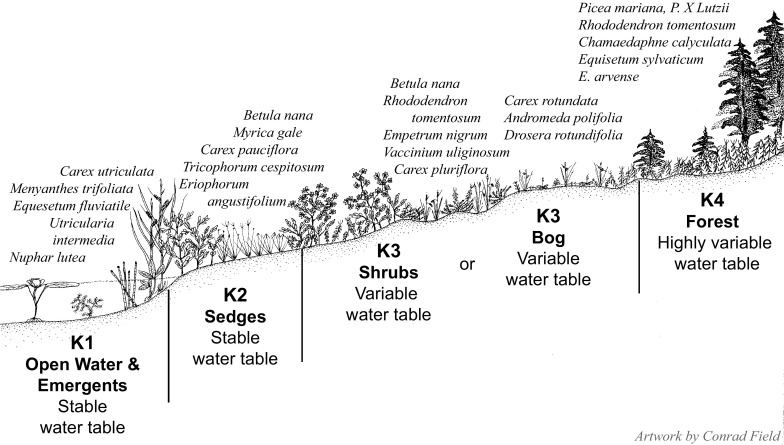
An idealized landscape cross-section showing the Hydrologic Components and common plant taxa found on the *Kettle* Geomorphic Component of the Cook Inlet Classification system

**Fig. 4:**
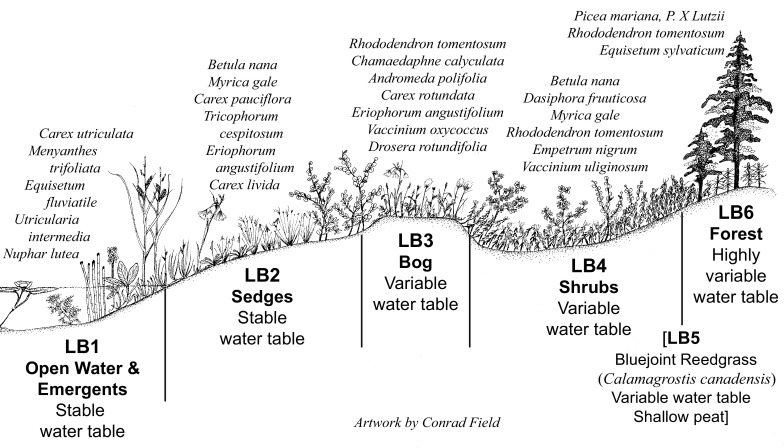
An idealized landscape cross-section showing the Hydrologic Components and common plant taxa found on the *Lakebed* Geomorphic Component of the Cook Inlet Classification system

**Fig. 5:**
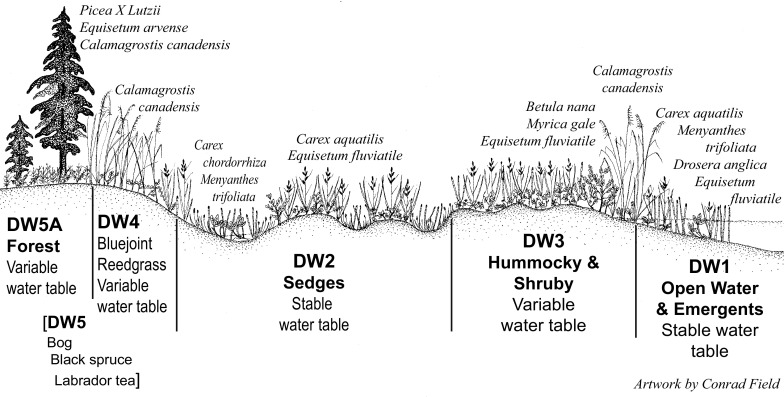
An idealized landscape cross-section showing the Hydrologic Components and common plant taxa found on the *Drainageway* Geomorphic Component of the Cook Inlet Classification system

Each Geomorphic Component of the CIC supports a somewhat different combination of pH, specific conductance, and seasonal variation in water levels (Fig. 2). For the Hydrologic Components, the numeric values assigned ranged from 1 to 6. Most Geomorphic Components had Hydrologic Components similar in character to those of *Kettles*, which were assigned Hydrologic Component values ranging from 1 to 4 (Fig. 3). These values are reflected in the vegetation found within this wetland type. For example, sites classified as the Geomorphic Component *Kettle* (*K*), with a Hydrologic Component = *1* (i.e. mapped as *K1*), support open ponded water with emergent plants such as yellow water-lily (*Nuphar lutea* (L.) Sm.) and water horsetail (*Equisetum fluviatile* L.). In contrast, sites classified as *K4* are in *Kettles* that frequently support a forest of black spruce (*Picea mariana* (Mill.) Britton, Sterns and Poggenb.) over an understory of Labrador tea (*Rhododendron tomentosum* Harmaja) (Fig. 3; Online Resource). The two extra hydrologic classes (*5* & *6*) were required in the Geomorphic Components *Drainageways* and *Lakebeds* because peatlands form more extensive complexes on these landforms. One of the extra Hydrologic Components represents bogs, which often occur as small recharge mounds within a larger expanse of fen vegetation, or as forested margins on relict glacial drainageway features *(LB3* in *Lakebeds* & *DW5* in *Drainageways*). The bog class is supported because a fundamental dichotomy between bogs and fens has long been recognized in peatland classification (Du Rietz 1949; Sjörs 1950; Glaser et al. 1990; Keimowitz et al. 2013). The other Hydrologic Component unique to *Lakebeds* and *Drainageways* (*LB5* on *Lakebeds* & *DW4* in *Drainageways*) (Figs. 4, 5) represents zones dominated by blue-joint reedgrass (*Calamagrostis canadensis* Michx. P. Beauv.), a common grass reported to be a keystone species in wetland-stream interactons in the CIB (Whigham et al. 2012). Both of these additional components can be distinctive over extensive areas on these larger peatland complexes.

For mapping purposes, within any Geomorphic Component the values for the Hydrologic Component can be combined to name a mapping unit. For example, the mapping unit *K32* indicates a polygon mapped in a *Kettle* peatland with a mixture of Hydrologic Components *3* and *2* at a scale too fine to be delineated separately at the nominal mapping scale. The first-named Hydrologic Component covers a greater area of the polygon. Polygons, rather than wetlands, were classified because several different peatland types have frequently coalesced into extensive complexes covering thousands of hectares (Online Resource).

### Plant prevalence index

**Fig. 6:**
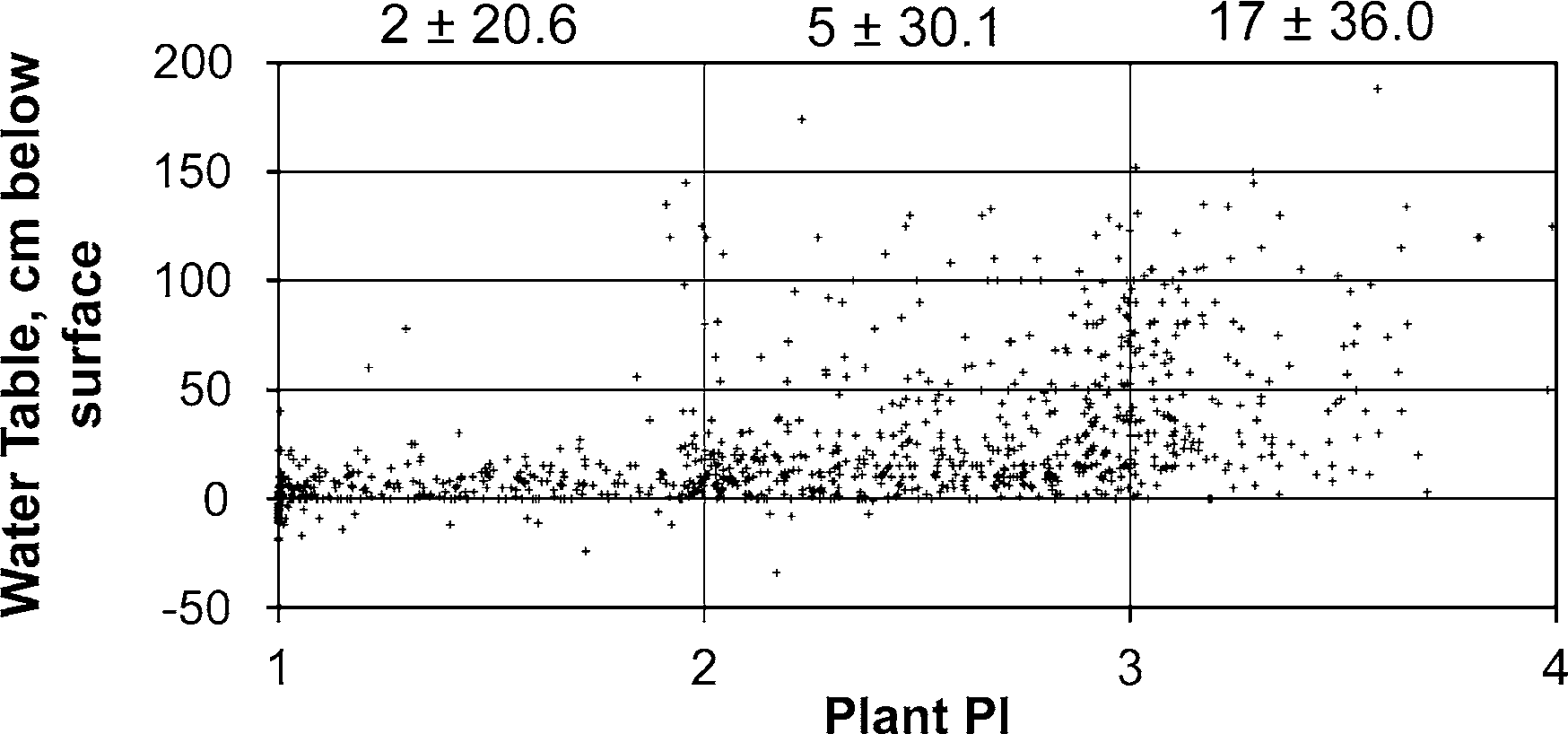
Plant prevalence index (PI) compared to estimates of the depth to the water table at 957 plots across CIB. *Negative values* indicate water above the surface. *Numbers along the top* are the median and standard deviation (cm) of the water table estimates for each corresponding range of PI values

Sites characterized by low PI values are more likely to support water levels that remain close to the land surface, whereas sites with higher PI values are associated with deeper and more variable water levels (Fig. 6). Plots with a PI between 1 and 2 are those supporting a predominance of wetland obligate plants (occur in wetlands > 99 % of the time) and plants that occur in wetlands more than 67 % of the time (taxa classified as *Facultative*-*Wet*). The median water level in these plots is close to the surface (2 cm), and they typically exhibit a lower variability in water levels throughout the year (s.d. = 20.6 cm) than plots scoring between 2 and 3 (5 ± 30.1 cm). Plots with a PI value of greater than three typically have a water level that is even deeper below the surface (median = 17 cm) and exhibits greater variability (s.d. = 36.0 cm) (Fig. 6).

### MRPP

All four classification schemes produce non-random groups of plots according to the MRPP (all with *P* < 0.001). The LLWW system by itself produced the lowest chance-corrected within-group agreement (*A*) in both procedures (*A* < 0.05). The Hydrologic Components of the CIC alone produced a relatively low *A* (0.06); as did the Geomorphic Components alone (*A* = 0.05). NWI and NWI + LLWW do not produce a within-group agreement greater than 0.1 in the procedure using PI, pH, and SC. However, the NWI system produces relatively high within-group similarity in the procedure using DCA axis scores (*A* = 0.13), as does NWI + LLWW (*A* = 0.18). In contrast the combined Hydrologic and Geomorphic Components of the CIC system produces the highest within-group agreement in both the procedure using PI, pH, and SC (*A* = 0.12), and the procedure using DCA scores from plant cover data (*A* = 0.21) (Table 2).

**Table 2:** MRPP *A* scores for the four classification schemes in the two procedures

Classification system	MRPP	
	DCA	PI, pH, SC
NWI	0.13	0.07
LLWW	0.03	0.04
NWI+LLWW	0.18	0.10
CICHydro	–	0.06
CICGeo	–	0.05
CIC	0.21	0.12

DCA is the MRPP using the first three axis scores from a Detrended Correspondence Analysis of plant cover data. PI, pH, SC is the procedure using physical and chemical variables where PI is Prevalence Index, and SC is specific conductance. CICHydro uses only the hydrologic classes of the CIC, and CICGeo uses only the geomorphic classes

**Fig. 7:**
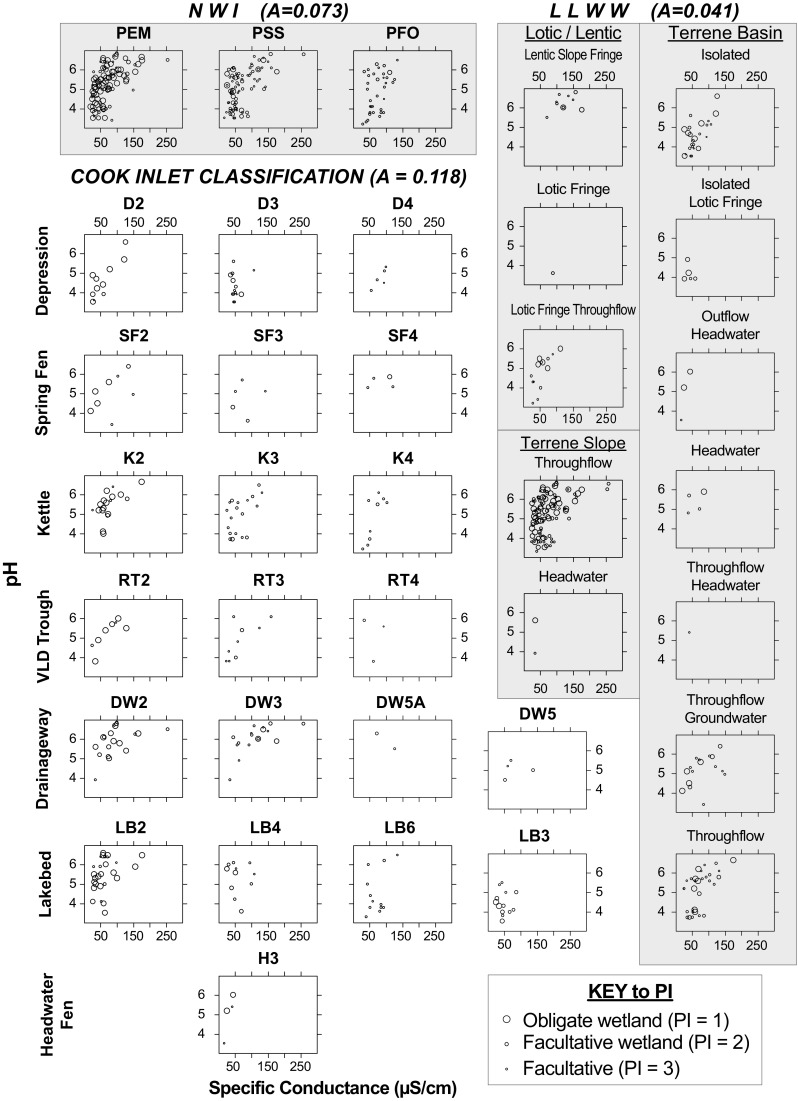
Specific Conductance, pH and PI for the three classification systems: Cook Inlet Classification (CIC), National Wetlands Inventory (NWI), and LLWW. The *size of the circles* is scaled continuously to plant Prevalence Index (PI), the key shows sizes corresponding to three important values of PI. CIC and NWI classes are arranged *left*-to-*right* from wetter to drier so that similar hydrologic classes are arranged in columns

The CIC plots are distributed evenly among the three NWI classes. These three classes (Palustrine emergent (PEM), shrub-scrub (PSS), and forested (PFO)) produce groups of plots characterized by similar values for PI, but plots within these groups exhibit a wide range of values for pH and SC (Fig. 7). In the LLWW system, most plots fall into five of the twelve classes (Fig. 7). Two of the classes, Lentic Slope Fringe and Inflow Lotic Fringe, separate two groups of peatlands: one with high values for pH and SC and the other with lower values. The hydrologic factor Throughflow differentiates some plots based on water chemistry because Throughflow peatlands in the landform classes Terrene Slope and Terrene Basin both contain plots with the highest values for SC (Fig. 7). However, these classes do not separate plots with different ranges in values for PI or pH, and patterns in PI, pH, and SC are similar between Throughflow peatlands in both the Terrene Slope and Terrene Basin classes (Fig. 7). Flow path classes do not appear to strongly differentiate plots based on PI, pH, and SC within any single geomorphic class. For example, the three flow-path sub-classes (Inflow, Throughflow Groundwater, and Throughflow) within the Terrene Basin class all exhibit a wide range of values for at least two of the variables (Fig. 7).

Peatland plots are evenly distributed among the 21 CIC classes, each of which broadly exhibits similar values for PI, pH, and SC. Although substantial overlap is evident, plots classified within the same Hydrologic Component have similar PI values across Geomorphic Components (columns in Fig. 7), whereas plots within the same Geomorphic Component generally exhibit similar patterns in pH and SC (rows in Fig. 7). For example, values of PI are similar between K2 & D2, or K4 & D4, whereas pH and SC are lower on shrubby *Lakebeds* (*LB4*) than in shrubby *Drainageways* (*DW3*) (Fig. 7). Plots with higher values for both pH and SC tend to occur in *Drainageways* and those with lower values occur in *Depressions*, with those in *Kettles* showing intermediate values overall. An exception is bogs on *Lakebeds* (*LB3*) which exhibit a relatively wide range in values for PI (Fig. 7).

## Discussion

Peatlands form autochthonously though the accumulation of organic matter, and as a result they likely exhibit different responses to environmental factors than that of non-peat forming wetlands in which the vascular plants are directly rooted in mineral soil. The accumulation of dead organic matter within raised bogs, for example, forms peat mounds that create a new set of hydrological flow conditions that did not previously exist at a site (Glaser and Janssens 1986; Siegel et al. 1995; Glaser et al. 1997). The CIC therefore produced greater within-group agreement because the hydrogeologic factors of the CIC system are more directly related to the fundamental ecological controls of oligotrophic peatlands than the more generalized factors used by the NWI, LLWW, or NWI + LLWW systems. The hydrologic factor of water level variation appears to be the most important control, similar to the findings of Foster et al. (1988), who discovered that surface patterns and peat accumulation rates in patterned fens in eastern Canada were primarily controlled by water table elevations. In northern Minnesota, moisture tolerance controlled the arrangement of plots in a gradient analysis (Glaser 1992). In in northern Sweden, (Waddington and Roulet 2000) found that methane production and carbon cycling were related to topographic position, which in turn is related to soil moisture. Glaser et al. (2004a) found that species richness of both vascular plants and bryophytes declined in a nearly linear relationship with increasing water level on the Hudson Bay Lowland, the most extensive peatland complex on Earth.

In the CIB, water-level variation may be even more important in creating different peatland types because patterns of variation may be enhanced by an uneven distribution of seasonal precipitation. In contrast to continental regions, where summer convective storms provide an equable distribution of precipitation throughout the growing season, peatlands in the CIB begin the season with a high water table following the flush of spring snowmelt and then must adjust to falling water levels during the driest months, which immediately follow. The wet season does not begin until late summer and early fall with the deepening of the Aleutian Low pressure system (climate diagrams in Fig. 1; Online Resource). The long lag between substantial recharge from spring snowmelt and the onset of fall rains regularly allows for a period of water level drawdown during the short growing season in CIB.

The classes identified by the CIC system are characterized by high within-group similarity in PI, a proxy for water level variation. Similarly, the three plant life-form classes used in the NWI system are likely related to water level variations, and it is not surprising that this classification system produces the next best within-group agreement. In contrast, the hydrologic classes of LLWW, which are defined by inferred water flow-path, produce low within-group similarity. The low similarity produced by the LLWW system agrees with findings of other investigations (*cf*. Shaffer et al. 1999). Although Cole et al. (1997, 2002) found similarities in hydrological conditions among flow-path classes within the state of Pennsylvania (USA), within-class similarity was low when the classes were extended across a wider region (Cole et al. 2008), or applied across the continent in the state of Oregon (Cole and Brooks 2000). Morrice et al. (2008) found that a similar classification system using flow path as a hydrologic factor did not define hydrologically distinct groups of Great Lakes coastal wetlands. These classification systems based on flow-path did not produce distinct groups in some regions likely because groundwater flow paths can vary, or even reverse, over time within the same wetland (Siegel and Glaser 1987; Siegel et al. 1995; Spence et al. 2011). Although shallow flow paths in peatlands can be assigned using the presence or absence of inlet and outlet streams, these relatively small streams may have little effect on the overall hydrodynamics in a peatland (Spence et al. 2011).

The seven specific landform names of the CIC system produce greater within-group agreement than the geomorphic factors employed in NWI, LLWW, or NWI + LLWW. Geomorphology should be related to water chemistry as long as the hydrogeologic setting is understood (Weber 1902; Glaser et al. 1997, 2004). However, the NWI uses only a single geomorphic factor to classify freshwater peatlands (Palustrine) and the physiognomic classes produce low within-group similarity based on the chemistry variables. These limitations are serious because different species within each life-form class can exhibit wide ecological tolerances with respect to pH and calcium concentration (Sjörs 1950; Glaser 1992). For example, whereas the shrub sweetgale (*Myrica gale* L.) will occur only on minerotrophic fens and not on ombrotrophic bogs, the shrub Labrador tea (*Rhododendron tomentosum* Harmaja) is found on both bogs and fens (Glaser 1992).

When applied to peatlands in the CIB, the LLWW system uses five landform types with respect to geomorphology, and these types have moderately similar ranges in water chemistry. However, the classes of the LLWW exhibited low within-group similarity overall, a result that agrees with findings of other investigations. For example, the LLWW did not produce groups of wetlands with similar ranges of water chemistry in the state of New York, USA (Azzolina et al. 2007). In addition, a classification system employing similar geomorphic classes also produced low within-group similarity with respect to the water chemistry of coastal wetlands in the Great Lakes region of North America (Morrice et al. 2008). Used alone, the Geomorphic Components of the CIC system produce greater within-group agreement than the combined geomorphic and hydrologic classes of the LLWW. Interestingly, the NWI system produces higher within-group agreement than the Hydrologic Components of the CIC system when the later are used alone in the analysis employing the variables PI, pH, and SC. This higher agreement is perhaps due to the fewer groupings of the NWI and the overriding ecological importance of water level variations (as indicated by PI) over chemistry (specifically pH & SC). The physiognomic classes of the NWI system are related to water level variation, but the Geomorphic Components of the CIC system require combination with its Hydrologic Components (which are related to water level variation) to achieve high within group similarity.

The importance of water level variation in producing within-group similarity is further demonstrated by the results of the MRPP using the combined NWI + LLWW classification system. In the procedure using PI, pH, and SC, the increase in within-group agreement of NWI + LLWW is small over either the NWI or LLWW systems by themselves. By itself, the LLWW system produces the lowest within-group agreement. The higher within-group agreement produced by the NWI system by itself is likely due to the strong relationship between water-level variation and its physiognomic classes. When the NWI and LLWW systems are combined, the small increase in within-group agreement suggests that the physiognomic classes of NWI are the primary driver of the increase, probably because the weak relationship between water-level variation and the LLWW classes imposes a limit on any potential increase in within-group agreement. By contrast, combining the Hydrologic and Geomorphic Components of the CIC produces a synergistic increase in within-group agreement. A system based on a combination of similar components may produce equally high within-group similarity across the region of boreal peatlands.

The relatively high within-group agreement produced by the combined Hydrologic and Geomorphic Components of the CIC shows that classes detectable on remotely-sensed imagery can better separate wetlands based on their response to fundamental drivers of ecosystem function. National-scale classification systems, in contrast, probably lack the resolution necessary to match the within-group agreement captured by any regionalized system. Morrice et al. (2008), for example, determined that a classification system devised for coastal wetlands around the North American Great Lakes performed better than a standard classification system that relies upon flow paths and broad landforms. Their system, which was based on a ratio between seiche and tributary hydrodynamics, was a better predictor of chloride concentration and variability than were the classes of the national system, which were based on flow path and landform. Chloride is an indicator of source water and of human disturbance, both fundamental controls on the ecological function of those Great Lakes wetlands. To the best of our knowledge, this study is the only other investigation that evaluated the performance of a regional wetland classification relative to that of a national system. Regardless of the factors, a regionally-specific system that produces high within-group similarity based on important ecological variables should be a useful tool for managers responsible for the maintenance of wetland ecosystem services.

One of the strengths of the CIC is its reliance on simplified proxies for the fundamental drivers of ecosystem function of wetlands in the CIB. In oligotrophic peatlands, specific conductance is strongly related to calcium concentration, which is generally the major cation balancing charge in most surface waters and has also been related to differences in vegetation (Vitt and Chee 1990). Fen indicator species cannot tolerate the low calcium concentrations found in ombrotrophic bog waters, whereas at high concentration calcium can be toxic to the normal development of the protonemata of *Sphagnum papillosum*, an ecologically important peatland moss (Boatman and Lark 1971; Clymo and Hayward 1982). Other solutes, especially nitrogen and phosphorus, may also limit *Sphagnum* growth at elevated concentrations (Bridgham et al. 1996). If limiting or toxic solutes are important, specific conductance alone may not be a reliable proxy. Although water level variability appears to be an overriding controlling factor in peatlands, the fundamental dichotomy of classifying peat landforms as either ombrotrophic bogs or minerotrophic fens is controlled by the general direction of the groundwater gradient: downward in bogs and upward or laterally in fens. The gradient is reflected by the chemistry of peatland pore waters, with ombrotrophic bogs having dilute acidic surface waters solely derived from precipitation and acidified by the production of organic acids from decaying *Sphagnum*, whereas waters from minerotrophic fens have higher cation concentrations and alkalinity due to groundwater inputs (e.g. Clymo 1983 Gorham et al. 1985; Siegel et al. 2006). The CIC includes hydrologic classes for bogs (e.g. *DW5* in *Drainageways* and *LB3* on *Lakebeds*). However, bogs may exhibit wide variations in water levels, because they can include a range of microtopographic variation including sedge-dominated lawns or low areas, intermediate shrubby hummocks or ridges, and higher forested crests (Sjörs 1948, 1963; Glaser and Janssens 1986). This variation within the bog classes, especially the common *LB3* class of the CIC, may partly explain why the hydrologic classes of the CIC examined alone produced slightly lower within-group agreement using PI, pH, and SC than did NWI by itself, which groups wetlands only according to life form. Although the fundamental division of peat landforms into bogs and fens can be identified using water chemistry and plant indicators (;Sjörs 1950; Glaser 1992), peatland classes based on differences in water level variation appear to form more distinct groups, at least where precipitation maintains a low diversity of bog landforms, as it likely does in the CIB.

### Research, management, and wetland assessment

The Cook Inlet Classification has been used in several investigations to stratify field sampling designed to relate stream ecosystem functions to the surrounding wetlands and landscape. The classes of the CIC guided the categorization of streams by geomorphic factors in the CIB, that could then be correlated to various indices of stream function. For example, Whigham et al. (2012) found that coarser-scale wetland classes did not explain the distribution of plant species along headwater streams as well as finer, reach-scale factors. They also found that *Calamagrostis canadensis* was potentially a keystone species in interactions between wetlands and headwater streams, reinforcing the use of this species to separate wetland classes within the *Lakebed* and *Drainageway* classes of the CIC. Although Walker et al. (2012) found that stream chemistry and temperature were strongly related to flow-weighted slope and not to differences in wetland class, King et al. (2012) reported that differences in wetland class were important drivers of stream condition, modulated by flow-weighted slope, which is comparable to the more widely used topographic wetness index (Sörensen et al. 2006). These factors explained the additional variance in both fish and macroinvertebrate community structure. In addition, Callahan et al. (2015) found significantly higher temperatures in salmon-bearing streams flowing through *Drainageways* compared to streams flowing through *Discharge Slopes*, two of the hydrogeologic classes defined by the CIC.

Local managers in the CIB are currently using the distinct Hydrologic and Geomorphic Components of the CIC to guide wetland functional assessment. The Hydrologic Components are used to assign principle hydrologic functions to peatlands by relating contrasts among different Components to categories of storage, recharge, and discharge. For example, peatlands with stable high water tables (Hydrologic Component less than 3) are rated as principally transmitting discharge. Geomorphic Components are used to rate peatlands based on contrasts in degree of hydrologic isolation and the transmissivity of underlying sediments. For example, isolated peatlands underlain with impermeable sediments (*Depressions*) are principally performing the hydrologic function of storage (Borough 2014).

In other hydrogeologic settings these contrasts among peatlands may differ. A mosaic of different bog and fen types, for example can develop over carbonate and silicaceous terrain depending on the local hydrogeologic setting, whereas in regions with much higher precipitation the development of raised bogs and patterned fens may be linked to the distance between the bounding rivers and rapid rates of glacial isostatic uplift (Glaser and Janssens 1986; Glaser et al. 2004). However, since water level variation and chemistry are primary sources of ecological variation among peatlands worldwide, a classification system based on these factors is likely to be useful to managers and scientists everywhere.

## Electronic supplementary material

Below is the link to the electronic supplementary material.

The online resource, entitled *Wetlands and Climate*, is a 36” × 44” color map sheet. The legend is in two parts: one part is a color matrix of the Hydrologic by Geomorphologic Components of the CIC and the other is composed of brief descriptions of each mapping component. Climate diagrams, which highlight seasonal and geographic patterns in temperature and precipitation, are shown for selected stations. Modeled precipitation is shown as isohyets and in shaded categories on an inset. Different geomorphic settings are shown as oblique aerial views in 3-D shaded-relief. Idealized landscape cross-sections show relationships among plant taxa and Hydrologic Components for each Geomorphic Component of the CIC system. Supplementary material 1 (PDF 23747 kb)
