# Supplementary material for: Evaluation of a wetland classification system devised for management in a region with a high cover of peatlands: an example from the Cook Inlet Basin, Alaska
Source: Wetl Ecol Manag. 2016 Oct 19;25(1):87–104. doi: 10.1007/s11273-016-9504-0 (PMC7115032; doi:10.1007/s11273-016-9504-0)
Supplement: Supplementary file 1 — The online resource, entitled Wetlands and Climate, is a 36” × 44” color map sheet. The legend is in two parts: one part is a color matrix of the Hydrologic by Geomorphologic Components of the CIC and the other is composed of brief descriptions of each mapping component. Climate diagrams, which highlight seasonal and geographic patterns in temperature and precipitation, are shown for selected stations. Modeled precipitation is shown as isohyets and in shaded categories on an inset. Different geomorphic settings are shown as oblique aerial views in 3-D shaded-relief. Idealized landscape cross-sections show relationships among plant taxa and Hydrologic Components for each Geomorphic Component of the CIC system. Supplementary material 1 (PDF 23747 kb) [file 11273_2016_9504_MOESM1_ESM.pdf]

SHEET 1. WETLANDS AND CLIMATE

KENAI PENINSULA

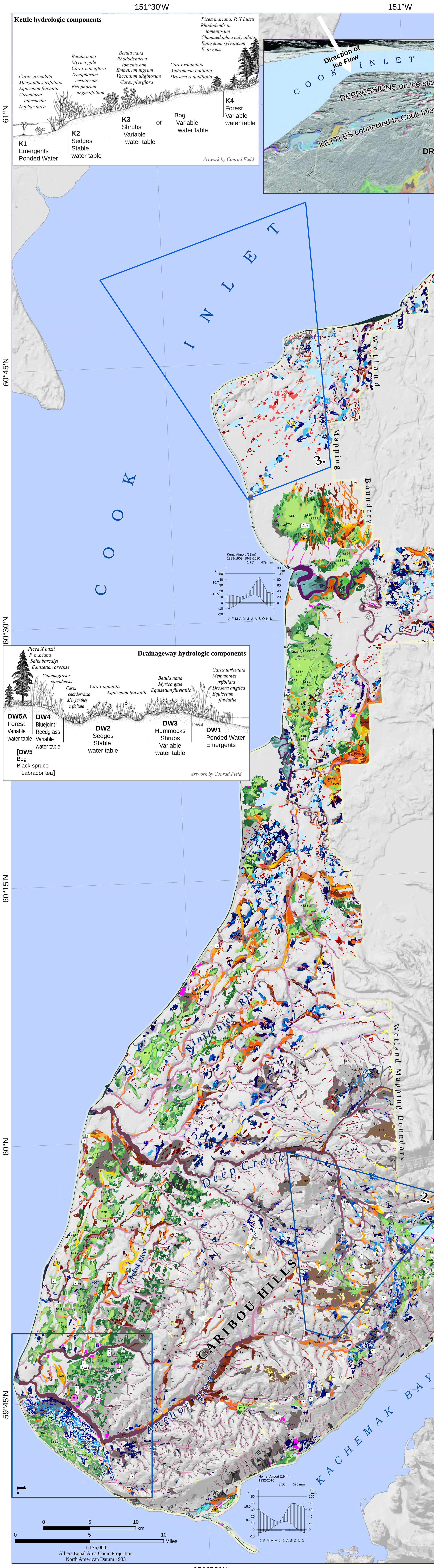

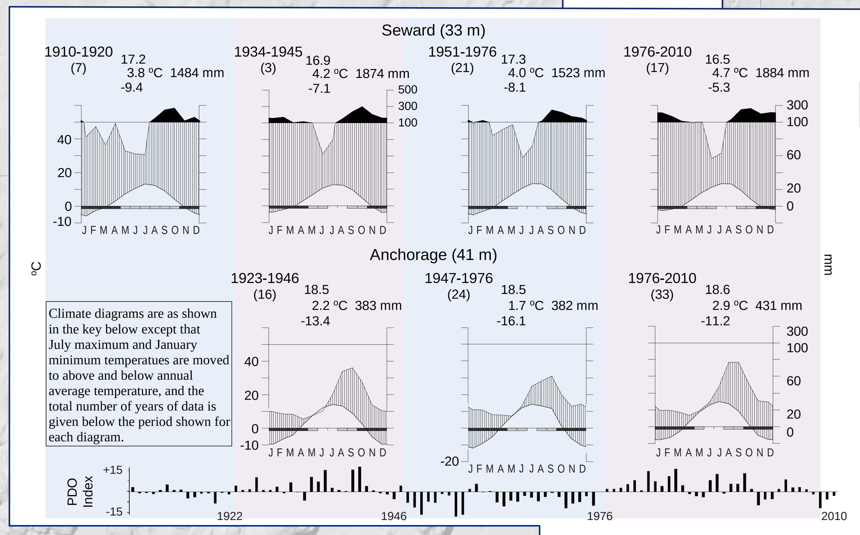

The Pacific Decadal Oscillation

The Pacific Decadal Oscillation (PDO) has pronounced effects on regional climate in the Cook Inlet Basin. The PDO is divided into two phases, warm and cool, which are related to an index of sea surface temperatures in the North Pacific Ocean (Mantua & Hare 2002). The climate diagrams shown above for Seward (top) and Anchorage (bottom) are constructed for each phase. Background shading shows cool (blue) and warm (pink) phases, and index values and ending dates for each phase are depicted by the bar graph across the bottom, except for 2010 which only shows the end of the data. Drier and cooler conditions prevail during cool phases, and wetter and warmer conditions previal during warm phases. Notice especially the wetter spring conditions in Seward during warm phases, the colder January minimum temperatures in Anchorage during the cool phase, and that July maximum temperature changes little between phases.

Bog formation on the Kenai Peninsula may be limited by the low amount of precipitation falling during the growing season, coupled with regular periods of drier conditions related to fluctuations in the PDO.

REGIONAL PRECIPITATION AND CLIMATE

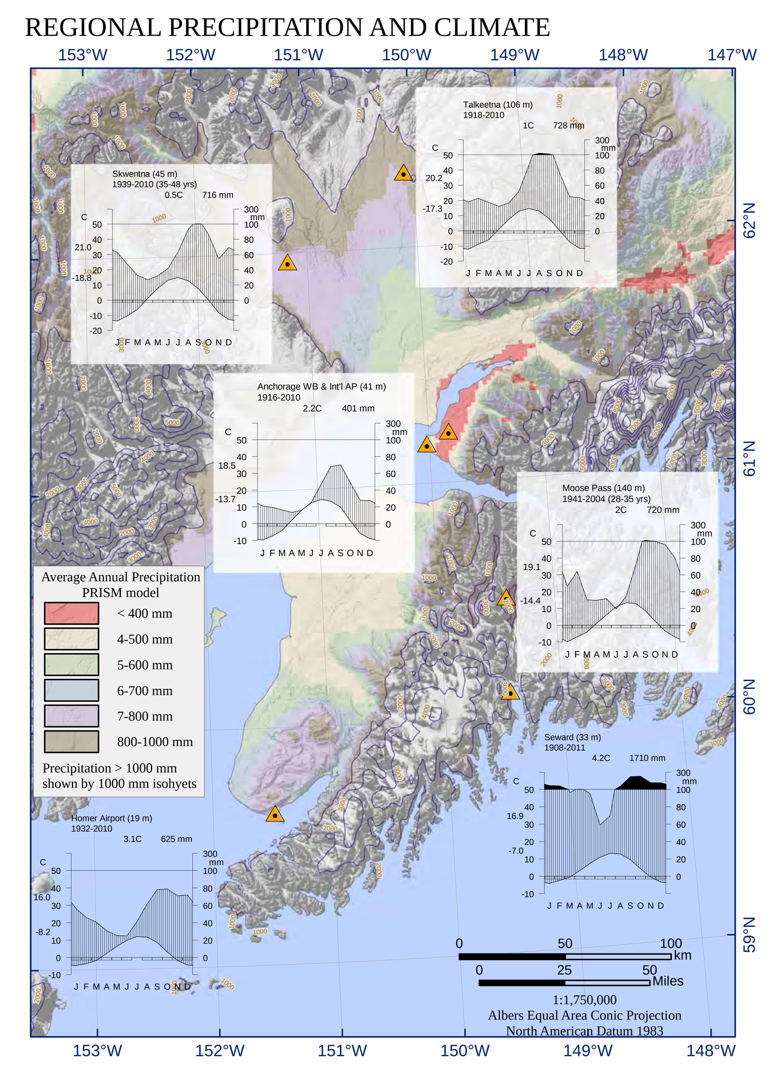

MATANUSKA-SUSITNA VALLEY

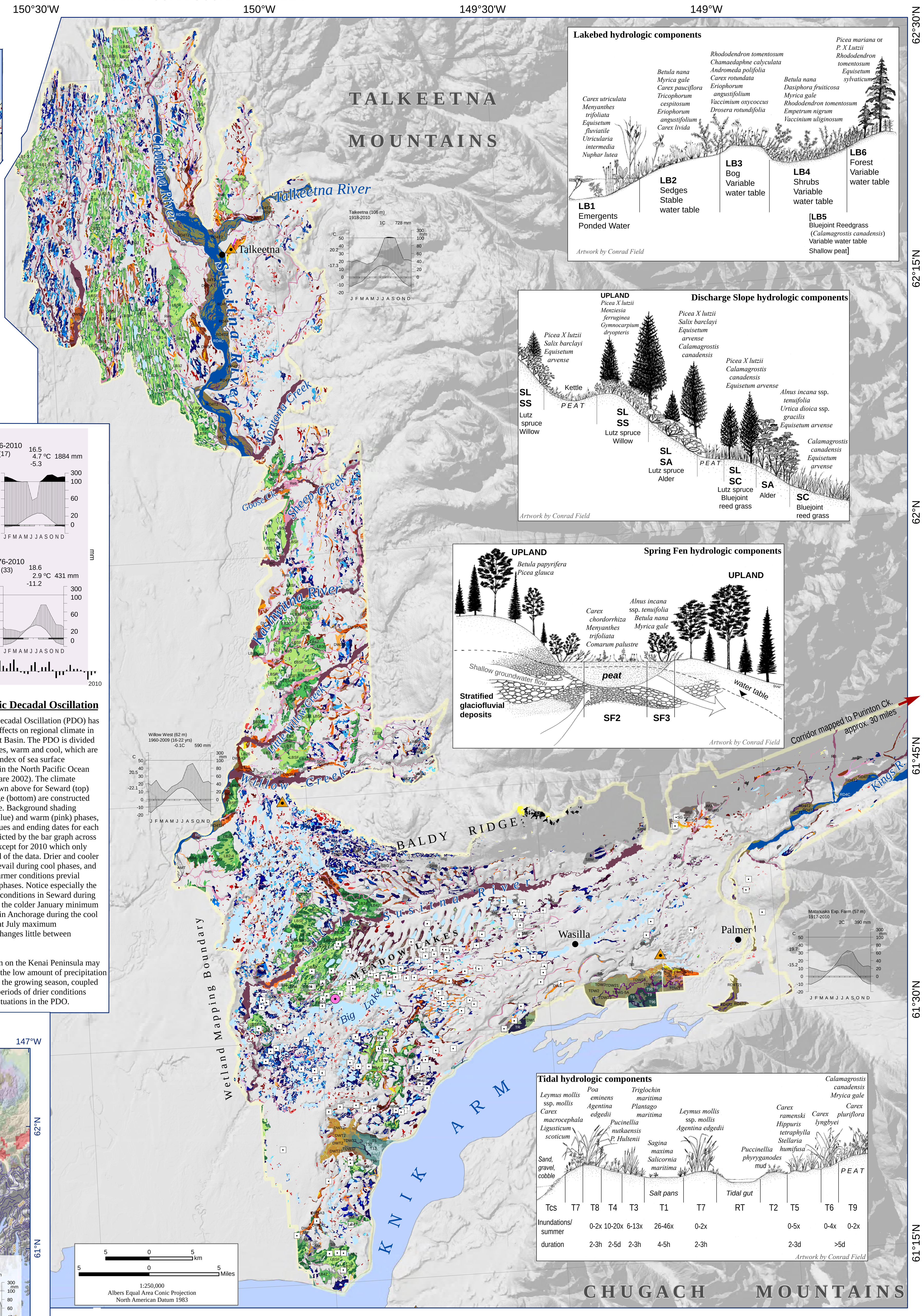

MAPPING COMPONENTS

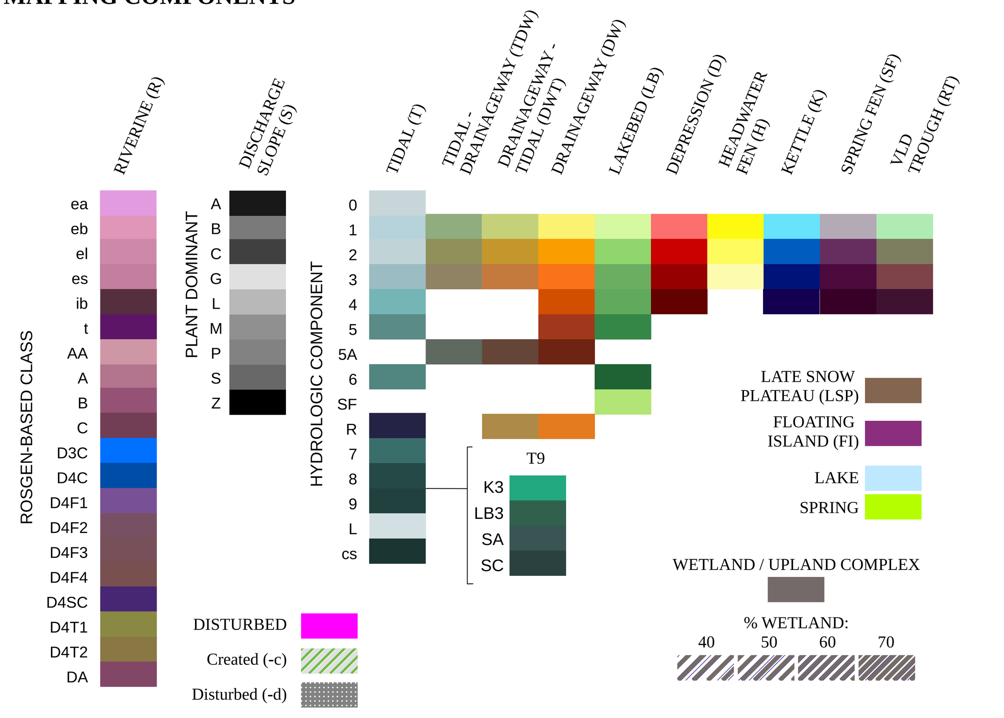

MAPPING COMPONENT DESCRIPTIONS

RIVERINE COMPONENTS

Rivers and streams and their associated valley bottoms
Based on Rosgen's (1996) morphological classification

E REACHES: slightly entrenched, stable, pool-dominated reaches within relict channels

Rea	Stream surface not discernable on 1:25,000 B&W aerial photography
Reb	Bank-full due to beaver dam, roads, logging debris or natural obstruction
Rel	Linear, sinuosity less than 1.2
Res	Sinuous, sinuosity greater than 1.2
Rib	Islands and bars
Rt	Tidally influenced reach
RAA	Cascade or waterfall
RA	High gradient (greater than 4%) riffle-dominated reach
RB	High gradient (2- 4%) riffle-dominated reach with scour pools
RC	Reaches with cut banks, point bars, and well-developed floodplains

D REACHES: Braided channels on glacial deposits: **D3** bed materials dominated by cobbles, **D4** dominated by gravel

RD3C	Channel of D3 reach
RD4C	Channel of D4 reach
RD4F1	Open water in D4 floodplain
RD4F2	Water table at or near surface in D4 floodplain, dominated by sedges
RD4F3	Variable water table in D4 floodplain, dominated by shrubs
RD4F4	Highly variable water table in D4 floodplain, forested
RD4SC	Side channel of D4 reach
RD4T1	Lower terrace of D4 reach
RD4T2	Upper terrace of D4 reach
RDA	Braided reaches on peat

DISCHARGE SLOPE COMPONENTS

Mineral soil wetlands at slope breaks. Classified by plant dominants

SA	Alder (*Alnus* Mill. spp.)
SB	Birch (*Betula* L. spp.)
SC	Bluejoint reedgrass (*Calamagrostis canadensis* Michx. Beauv.)
SG	White spruce (*Picea glauca* (Moench) Voss)
SL	Lutz spruce (*Picea X lutzii* Little)
SM	Black spruce (*Picea mariana* (Mill.) Britt., Sterns & Pogg.)
SP	Sitka spruce (*Picea sitchensis* (Bong.) Carr.) only mapped in Seward
SS	Willow (*Salix* L. spp.)
SZ	High elevation meadow, only on Baldy Ridge above Wasilla

TIDAL COMPONENTS

Influenced by at least the annual tidal cycle. Following zones defined by Vince & Snow (1984)

T0	Bare mud
T1	Saltpannes<br>Glasswort (*Salicornia maritima* Wolff & Jefferies) & pearlwort (*Sagina maxima* Gray)
T2	"Outer Mudflats zone 1" inundated 26-46x / summer<br>creeping alkaligrass (*Puccinellia phryganodes* (Trin.) Scribn. & Merr.)
T3	"Inner Mudflats zone 5" inundated 6-13x / summer<br>Goosetongue (*Plantago maritima* L.) & seaside arrowgrass (*Triglochin maritima* L.)
T4	"Outer Mudflats zone 2" inundated 10-20x / summer<br>Alkaligrass (*Puccinellia nootkaensis* (J. Presl) Fern. & Weatherby & *P. Hultenii* Swallen)
T5	"Outer Sedge Marsh" inundated 0-5x / summer<br>Ramensk's sedge (*Carex ramenskii* Komarov)
T6	"Inner Sedge Marsh" inundated 0-4x /summer<br>Lyngbye's sedge (*Carex lyngbyei* Hornem.)
T7	Beachrye (*Leymus mollis* (Trin.) Pilger ssp. *mollis*) plant community on storm berms
T8	"Riverbank Levee" & "Inner Mudflats" inundated 0-2 & 8-13x / summer<br>Pacific silverweed (*Argentina egedii* (Wormsk.) Rydb.) & largeflower speargrass (*Poa eminens* J. Presl)
T9	"Inner sedge marsh" inundated 0-2x / summer<br>manyflower sedge (*Carex pluriflora* Hultén)
TL	Lagoon
TR	Complex of components, often along tidal guts
Tcs	Barren sand, gravel, cobbles & boulders, at the storm line below bluffs

SPRING FEN COMPONENTS

Peatlands in ice-block depressions not connected by wetlands or surface water to navigable water and fed by strong ground water connections where precipitation is low

SF1	Ponded water
SF2	Water table at or near the surface, usually sedge-dominated
SF3	Variable water table, usually shrub-dominated
SF4	Highly variable water table, usually forested

VERY LARGE DUNE TROUGH COMPONENTS

Peatlands in low-lying areas between the very large dune features found only in the Meadow Lakes area of the Matanuska-Susitna Valley

RT1	Ponded water
RT2	Water table at or near the surface, usually sedge-dominated
RT3	Variable water table, usually shrub-dominated
RT4	Highly variable water table, usually forested

HEADWATER FEN COMPONENTS

High elevation peatlands at the headwaters of first-order streams

H1	Ponded water
H2	Water table at or near the surface, usually sedge-dominated
H3	Variable water table, usually shrub-dominated
H4	Highly variable water table, usually forested

DEPRESSION COMPONENTS

Peatlands in ice-block depressions not connected by wetlands or surface water to navigable water

D1	Ponded water
D2	Water table at or near the surface, usually sedge-dominated
D3	Variable water table, usually shrub-dominated
D4	Highly variable water table, usually forested

RELICT GLACIAL DRAINAGEWAY COMPONENTS

Peatlands in features once draining extensive glaciers

DW1	Ponded water
DW2	Water table at or near the surface, usually sedge-dominated
DW3	Variable water table, usually shrub-dominated
DW4	Shallow peat, dominated by bluejoint reedgrass (*Calamagrostis canadenis* Michx. Beauv.)
DW5	Bogs. Bog indicators (pH<4.2; [Ca]<2 mg/l; lack of fen plants) are masked by tephra on the Kenai Peninsula.
DW5A	Variable water table, forested
DWR	Complex of DW components, often a narrow watercourse fringed by shrubs and forest

RELICT GLACIAL LAKEBED COMPONENTS

Peatlands on features once covered by extensive ice-marginal or proglacial lakes

LB1	Ponded water
LB2	Water table at or near the surface, usually sedge-dominated
LB3	Bogs. Bog indicators (pH<4.2; [Ca]<2 mg/l; lack of fen plants) are masked by tephra on the Kenai Peninsula.
LB4	Variable water table, usually shrub-dominated
LB5	Shallow peat, dominated by bluejoint reedgrass (*Calamagrostis canadensis* Michx. Beauv.)
LB6	Variable water table, forested. When combined with LB3, a bog
LBSF	Complex of LB components, often a patterned fen with tree islands and strang-and-flark microrelief

KETTLE COMPONENTS

Peatlands in ice-block depressions connected by wetlands or surface water to navigable water

K1	Ponded water
K2	Water table at or near the surface, usually sedge-dominated
K3	Variable water table, usually shrub-dominated
K4	Highly variable water table, usually forested

TIDAL - DRAINAGEWAY COMPONENTS

Influenced by extreme spring tides where tidal range is exceptional.
Tidal influence dominates freshwater in TDW; Freshwater dominates in DWT.
Only mapped along Knik Arm

TDW1	Ponded water	DWT1
TDW2	Water table at or near the surface, usually sedge-dominated	DWT2
TDW3	Variable water table, usually shrub-dominated	DWT3
TDW5A	Variable water table, forested	DWT5A
DWTR	Complex of DWT components, a narrow watercourse fringed by shrubs and forest	

AMT	**ABANDONED MEANDER TERRACES along the Kenai and Fox Rivers**
LSP	**LATE SNOW PLATEAUS: Mineral soil wetlands over thin discontinuous till at high elevations on the Kenai Peninsula in the Caribou Hills**
FI	**FLOATING ISLANDS: Peat islands floating in lakes often dammed by human activity**
WU	**WETLAND / UPLAND COMPLEX: Wetlands comprise at least 30%. Actual percentage shown in the Matanuska-Susitna Valley by hatching:** % WETLAND: 40 50 60 70
	**DISTURBED: Wetland mapping unit not discernable due to human disturbance**

Wetland **created** by human activity, shown by a suffix **c** appended to any mapping unit

Wetland **disturbed** by human activity, shown by a suffix **d** appended to any mapping unit

LOCATION IN ALASKA

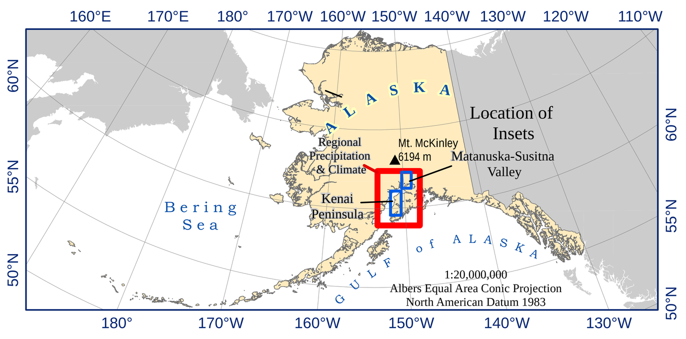

KEY TO CLIMATE DIAGRAMS

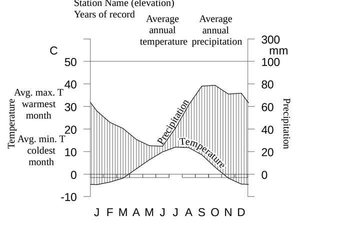

After: H. Walter & H. Leith 1960. *Klimadiagramm-Weltatlas*. G. Fischer, Jena.

SYMBOLS

- Location of plots used in MRPP analysis
- Climate Stations
- Peat coring sites

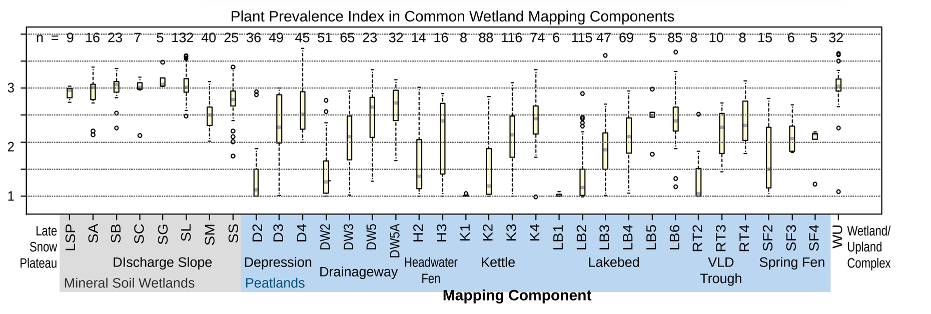

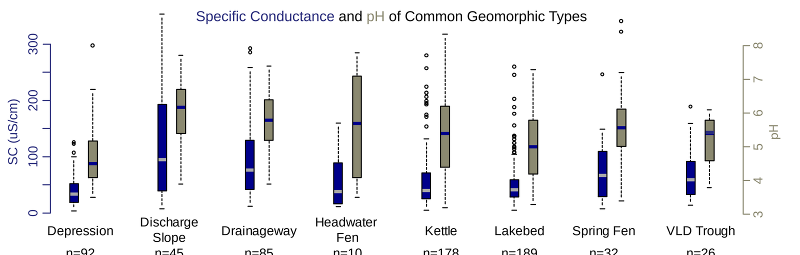

Prevalence Index (PI) is related to the position and variability of the water table. PI = 1 indicates a plot with only wetland obligate plants, plots with PI > 3 may not meet wetland criteria. Specific Conductance (SC) and pH show groundwater influence.
In the box plots, boxes enclose the first through third quartile; the gray bar is the median, and the whiskers extend to the last value within 1.5 times the inner quartile range. Values lying beyond 1.5 times the inner quartile range are plotted as hollow circles.
The number of samples for each map component is given across the top of the Prevalence Index graph.

NOTES: Wetlands were mapped using stereo-paired aerial photography at various scales: 1:12,500 around Homer, 1:24,000 on the Kenai Peninsula outside of Homer, and 1:18,000 in the Matanuska-Susitna Valley. Approximately 10-20% of wetland polygons have been visited. Wetlands on this map are displayed at a scale of 1:175,000 & 1:250,000 to convey a general sense of their realtive abundance and relationship to physiography. Visit cookinletwetlands.info to view more information, including metadata, and to download a .pdf file of this map, or other mapping files viewable in either ArcGIS or GoogleEarth.
Numbered insets on the Kenai Peninsula Map show simulated 3D models of representative glacial landscapes corresponding to areas outlined in blue.

Data Sources:
Climate data used to construct climate diagrams: Utah Climate Center, Utah State University: http://climate.usurf.usu.edu/products/data.php
Digital Elevation Model used in shaded relief maps: Alaska Geospatial Data Clearinghouse, US Geological Survey: http://agdc.usgs.gov/data/usgs/to_geo.html
LiDAR used in 3D landscape diagrams: Kenai Watershed Forum, Soldotna Alaska.
Precipitation contours: PRISM Climate Group, Oregon State University, http://prism.oregonstate.edu Map created 4 October 2011.

LITERATURE:
NJ Mantua and SR Hare (2002) The Pacific Decadal Oscillation. Journal of Ocenography:35-44.
Rosgen, D. and H.L. Silvey, 1996. Applied River Morphology. Wildland Hydrology Books, Pagosa Springs, Colorado, USA. 325 pp.
Vince, S.W. and A. Snow. 1984. Plant zonation in an Alaskan salt marsh: I. Distribution, abundance and environmental factors. Journal of Ecology 72: 651-667.

"Cook Inlet Wetland Classification". Michael Gracz, Kenai Watershed Forum, University of Minnesota Conservation Biology Program. gracz016@umn.edu 25 February 2014
